# Clinical and structural changes in reproductive organs and endocrine glands of sterile cows

**DOI:** 10.14202/vetworld.2020.774-781

**Published:** 2020-04-24

**Authors:** Evgeny Skovorodin, Ravil Mustafin, Svetlana Bogoliuk, George Bazekin, Valian Gimranov

**Affiliations:** Department of Morphology, Pathology, Pharmacy, and Non-communicable Diseases, Federal State Budget Educational Institution of Higher Education Bashkir State Agrarian University, Ufa, Russia

**Keywords:** cows, endocrine glands, morphology, ovaries, reproductive organs, sterility

## Abstract

**Aim:**

The study aimed to determine both macroscopic and microscopic changes in the reproductive organs of sterile cows.

**Materials and Methods:**

Careful clinical and gynecological examinations (using histological and histochemical methods) of organs of culled sterile cows, such as ovaries, oviducts, the uterus, pars anterior adenohypophysis, thyroid body, and adrenals, were performed.

**Results:**

It was found that 20% of the examined cows in the farms of the Republic of Bashkortostan had pathology of reproductive organs. Ovarian dysfunction was diagnosed in 31% of sterile cows. Histological and histochemical studies revealed that high atresia of all types of ovarian follicles is associated with hypofunction of the ovaries. This was related to stromal vascular dystrophy and was accompanied by atrophy of thecal endocrine elements, resulting in decrease of endocrine and generative function of ovaries.

**Conclusion:**

Essential elements of the ovarian dysfunction pathogenesis are disorders of the functional system “ovary-pituitary-adrenal-thyroid gland” and the abnormality of utero-ovarian relationships, which differ significantly during hypofunction, in case of follicular cysts, and in case of persistent corpora lutea. This difference in abnormalities of utero-ovarian relationships should be considered when developing diagnostic and treatment methods and determining preventive measures

## Introduction

To intensify animal breeding, modern digital technologies should be implemented [1,2]. However, to obtain quality products, a herd of highly productive cattle with optimal reproductive capacity and cows with long-term use, are required [3]. In the United States and several Western European countries, the annual rate of cow disposal, which results mostly due to cows’ sterility, is 34% [4]. The only method to compensate these losses is intensive introduction of first-calf heifers into seedstock herd; however, the quickest method is importing of bred heifers [5]. Bad welfare and exploitation of imported animals result in early culling of cows after two lactations [6] as well as inability to obtain healthy offspring from them for breeding, to increase productivity [7,8]. Therefore, sterility leads not only to economic losses but also biological damage by reducing the cattle’s genetic potential [9,10].

The main cause of sterility in cows is diseases of reproductive organs [10-16]. Thus, a careful study on pathomorphism of the reproductive system will allow to objectively defining the nature of this pathology [17-25].

Reproductive organs of 1113 cows of a meat processing facility were studied. The results revealed following pathological changes in the ovaries of 6.02% cows and in the structures surrounding the ovaries of 5.21% cows: Follicular cysts (1.88%), corpora lutea cysts (2.51%), paraovarian cysts (0.72%), bursa oviduct cysts (0.18%), periovarial adhesions (0.27%), granulose cellular tumors (0.27%), and hemangioma (0.09%). In 0.81% of cows following pathological changes were found in the oviduct: Hydrosalpinx (0.36%), mesosalpingitis and adhesions (0.09%), and salpingitis (0.45%) [24].

Rathore *et al*. [25] conducted a pathological study of the reproductive organs of 390 cows. Pathohistological analysis of 129 samples collected from cows with signs of pathology revealed following pathologies: Oophoritis (3.20%), acute salpingitis (0.64%), chronic salpingitis (2.56%), acute endometritis (0.64%), subacute endometritis (1.92%), chronic endometritis (18.58%), and metritis (3.84%). Owhor *et al*. [15] concluded that salpingitis is the common cause of infertility among both humans and animals.

Histological and electron microscopic analysis of cow ovaries revealed a high number of abnormal ovarian follicles [26]. Hence, fertilization of oocytes obtained from such cows differs significantly [27]. A positive relationship exists between the size of the ovarian reserve and the number of antral follicles in the ovary. In beef cows, the number of antral follicles increases until they are 5 years old and decreases as they become older. This may indicate that a decrease in fertility of beef cows due to reduction in ovarian reserve, can begin earlier than it was thought before [28].

A histological study by Vargas *et al*. [29] on the ovarian corpora lutea of slaughterhouse cows indicated that the nucleoplasmic ratio in luteal cells decreased with an increase in pregnancy time and the relative volume of stroma increased.

Follicular cysts result from the inability of a mature follicle to ovulate on time in the estrous cycle. Further, changes in endocrine glands lead to the persistence of follicular ovarian cysts [12].

However, according to the literature, no comprehensive studies focused on the functional and morphological analysis of the reproductive system as a whole have been conducted. This study aimed to determine the clinical and morphological manifestations of the pathology of reproductive organs and endocrine glands, regulating the reproductive function in the course of hypofunction of the ovaries, persistent yellow body, and follicular cysts of ovarian glands, in highly productive imported cows.

## Materials and Methods

### Ethical approval

All the experiments were performed in accordance with the legislation of the Russian Federation and considering the European Convention for the Protection of Domestic Animals (Council of Europe-ETS No.125-European Convention for the Protection of Domestic Animals).

### Study location

The study was conducted in the laboratories of morphology, pathology, pharmacy, and non-communicable diseases of the Bashkir State Agrarian University, on dairy farms of the Republic of Bashkortostan, and in meat processing facilities.

### Methods of clinical and anatomical research

Rectal and pelvic examinations of 283 infertile cows (Russian black pied breed) were performed on farms to study reproductive function of cows. Reproductive organs of 53 infertile cows from a government approved slaughterhouse were examined after slaughtering. These reproductive organs were taken for a microscopic examination. The ovaries were cut into plates 5mm thick for anatomical examination. The uterus was opened and pieces were cut at the anterior fornix and the caudal part of uterine horns for a macroscopic examination.

Further, cows’ uterine tubes patency or oviduct patency was assessed. The method involved filling the oviducts with air under excessive pressure and assessing the patency by the pressure drop. The oviduct was separated from the uterus and dipped into a liquid in an experimental dish. Simultaneously, air was introduced through the oviduct funnel. Then, the patency was estimated by the amount of air leaving the uterine extremity and by the degree of pressure drop [30].

### Methods of histological and histochemical study

Liquid nitrogen, Carnoy’s fluid, and 10% aqueous formalin solution were used for tissue fixation. After eviscerating the pituitary gland, it was cut into two halves. One half was fixed using Cenker’s liquid (sulem mixture) and the other half using Bouin’s solution. Sections of the central parts of thyroid lobes were fixed in Carnoy’s fluid and 10% aqueous formalin solution. Adrenal glands and ovaries were cut into plates and fixed.

The plates of tissues were paraffinized and sectioned according to the standard practice. These sections were stained with hematoxylin and eosin and using the methods described by Pappenheim, Van Gieson, and Mallory; by Bilshovsky-Gross, Einarson, Brachet, Felgen, and Rossenbec; and by McManus and Steedman. In addition, sections were frozen using a freezing microtome and stained with alcohol solutions of Sudan III and Sudan black B, according to the method described by Romeis. The pituitary gland was stained with paraldehyde fuchsine orange as per the method described by Halmi [31]. Furthermore, the McManus and Schick tests were performed to detect glycogen and neutral glycoprotein.

Pearce’s formula [32] was used to determine the strength of the following oxidation-reduction enzymes in the frozen sections: Succinate dehydrogenase, lactate dehydrogenase, nicotinamide adenine dinucleotide reduced dehydrogenase, and nicotinamide adenine dinucleotide phosphate reduced dehydrogenase. Strengths of alkaline phosphatase and acid phosphatase in cryostat sections were determined using the method described by Burstone [33] respectively.

### Statistical analysis

Student’s t-criterion was used to determine the statistical validity of the indicator. Due to a relatively small number of groups and deviations of variables from normal distributions, a Kruskal–Wallis non-parametric analysis of variance test was performed using licensed software package Statistica 10 (Tibco Software Inc., CA, USA.). The level of significance was set at p<0.05.

## Results

### Analysis of the reproductive function of cows and the spread of ovarian diseases

Health abnormalities were noted in 34% of cows imported to the Republic of Bashkortostan. Clinical study revealed the following pathologies: Atony of the rumen, limb diseases, and signs of osteodystrophy. Biochemical analysis revealed intensive protein and mineral metabolism. Further, nearly total carotene deficiency was noted. Decompensated acidosis was found in 70% of blood samples. Hence, 6% of cows died and 4% were forcibly killed.

Diseases of reproductive organs were detected in 129 examined cows in farm number 1 (Table-1).

**Table 1:** Results of clinical and gynecological examination of cows.

Diagnosis	Number of animals in (%)
Morbid labor	8 (6.2)
Postpartum hypocalcemia	7 (5.4)
Metritis	2 (1.6)
Retention of placenta	15 (11.6)
Endometritis	31 (24)
Subinvolution of uterus	20 (15.5)
Hypofunction of the ovaries	32 (24.8)
Persistent corpora lutea	4 (3.1)
Ovarian cyst	7 (5.4)
Pathology of oviducts	3 (2.3)
Total	129 (100)

A total of 40% cows had uterine diseases, especially after calving. The pathology of oviducts was clinically diagnosed in 2.3% of infertile cows. Ovarian diseases were diagnosed in 33.3% of infertile cows: About 5.4% cows had follicular cysts, 3.1% had persistent yellow body, and 24.8% had hypofunction of the ovaries.

Hypofunction of the ovaries-ovarian was noted in 73% cows with a background of diseases of the respiratory system, cardiovascular system, metabolic disorders, and diseases of the limbs (Table-2). Such animals experienced long-term anaphrodisia, atrophy of ovaries, and the uterus.

**Table 2:** Spread of gynecological diseases in farm no. 2.

Total number of infertile cows	Hypofunction of the ovaries	Hidden sexual cycle	Persistent corpora lutea of the ovary	Endometritis	Cervicitis
154	112	24	13	4	1
100%	73%	16%	8%	2.3%	0.7%

**Figure-1:**
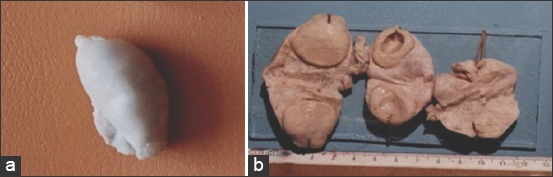
Ovaries of infertile cows. (a) Hypofunction of the ovaries (b) corpora lutea of cows: During pregnancy time, cystic, persistent.

Cows with hypofunction of the ovaries-ovarian and those culled due to sterility had small ovarian glands (size, 3-1.5cm; volume, 9-28 cm^3^). They were round, oval, elongated, flat, and bean shaped (Figure-1a). Follicles found in incisions had a well-defined white coat or they were slightly collapsed, and their parametrium was yellow. One to four yellow and red corpuscles were found on the incision surface. Atotal of 30% cows with ovarian diseases had chronic catarrhal endometritis.

Diseases of oviducts, as well as pathology of paraovarian structures, are macroscopically detected more often in killed animals: Hydrosalpingitis (6.9%), ovarian paris cysts (3.4%), ovario-bursal adhesion (37.8%), cysts and adhesions of the meso-ovarium and mesosalpinx (17.0%), ovary medulla cysts (networks) (3.4%), and serous inclusion cysts on the ovarian gland surface (6.9%).

Gross obstruction of oviducts was found in 54% of cows (unilateral obstruction: 46% cows and bilateral obstruction: 8%) and relative obstruction (uterine tubes stenosis) was diagnosed in 46% of cows. However, an increase in the organ patency was observed in 4% of cows during uterus subinvolution.

**Figure-2:**
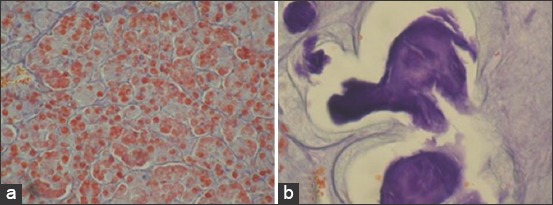
Adenohypophysis of a cow with hypofunction of the ovaries. Method of Halmi. (a) Acidophils predominance 100× (b) cysts in the central area of the gland with basophilic colloid 40×.

Some cows with hypofunction of the ovaries had small cysts in the center of the adenohypophysis which was confirmed histologically (Figure-2a). Thyroid glands were slightly enlarged. In the adrenal cortex of some cows a radial mass of yellow color, identified as amyloid, was found. Atotal of 50% cows who had been infertile for a long time had well-marked signs of protein and fatty dystrophy and hemodynamic disorders in the liver.

Persistent corpora lutea in the ovary protruded above the ovarian gland surface in the form of a fungus (Figure-1b). Sometimes a large cavity was found in the ovary, and simultaneously, uterus endometritis was often diagnosed. Dystrophic changes in the liver were rarely detected.

A total of 60% cows had follicular cysts with inflammatory processes in the uterus and oviducts. All cows had more than two follicular cysts in the ovaries, with the maximum number not exceeding three. Follicular cysts were found in the right ovary in 50% of cows, in the left ovary in 30% of cows, and in both ovaries in 20% of cows.

### Micromorphology of the reproductive organs of cows with ovarian diseases

Fibroplastic processes with atrophy of hormone-active structures were observed in the connective tissue and vascular network of ovaries of cows with ovarian dystrophy. In ovaries with persistent corpora lutea, the connective tissue architecture slightly differed from that of cows with a balanced sex cycle. The development of follicular ovarian cysts led to cortical substance thinning and to a decrease in the location density of non-luminal follicles.

Small follicles disappear when changes in the oocyte begin. Then, follicular membrane disappears and is replaced by a tender connective tissue. The ovarian dysfunction is accompanied by mass elimination of such ovarian follicles. In this case, oocyte apoptosis occurs quickly. Simultaneously, the follicular epithelium is preserved even after a germ cell disappears.

The two established morphogenetic mechanisms of luminal follicle atresia are: Obliteration of the cavity with connective tissue and filling cavity with luteinized follicular epithelium. Morphogenesis differs significantly in both mechanisms. The atretic process during hypofunction of the ovaries is characterized by the severity of dystrophic, necrotic, and sclerotic processes of generative and endocrine organ elements. The follicle cavity is neither filled-up completely nor quickly. However, large areas of hyalinized connective tissue can be observed.

### Morphology of endocrine glands of cows with ovarian pathology

The main adenohypophysis volumes, represented by chromophobes, in cycling C\cows, cows with persistent corpora lutea in ovaries, and cows with follicular cysts, were 65%, 68%, and 69%, respectively. Cycling cows and cows with follicular cysts had larger number of basophilic cells (20% and 21%, respectively) than cows with persistent corpora lutea in ovaries (14%; the difference was significant when p<0.05).

The number of chromophobes decreased during ovarian atrophy (39%). The number of acidophils was significantly higher than the number of basophils (38%). The organ stroma volume was large. Zonal distribution of different cell types in adenohypophyses of cows with hypofunction of the ovaries was not clearly expressed. Large pseudofollicles filled with polychromatophil colloid were found more often in the C\central zone of pituitary gland (Figure-2b).

Cytological examination of the distal adenohypophyses of cows with hypofunction of the ovaries revealed a low functional activity of somatotrophic hormone cells and a high functional activity of lactogenic hormone cells. Thyroid-stimulating hormone (TSH) and adrenocorticotropic hormone (ACTH) cells of cows with hypofunction of the ovaries slightly differed in structure compared with those of cows with balanced sex cycle. Acidophiles, basophils, and chromophobes of cows with corpus lutea, and follicular cysts in the ovaries were in the active stages of the secretory process, resulting in a high level of gonadotropic hormones in these cows. Pituitary dysfunction is proved by formation of large cavities in the central zone. The glandular tissue of cows with a persistent yellow body is characterized by dystrophic changes and apoptosis.

**Figure-3:**
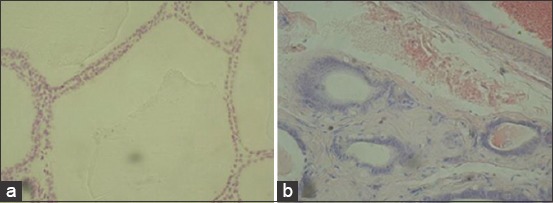
The thyroid gland of infertile cows. Staining hematoxylin and eosin. (a) Hypothyroidism 100× (b) fibrosis of the stroma 100×.

Thyroid glands of cows with different states of the reproductive system have relative volumes of colloid and epithelium, indicating a higher activity of the organ of cows with corpus lutea and cows with follicular cysts. In cows with ovarian hypofunction, sclerosis of the thyroid stroma was expressed, which significantly reduces the function of this organ (Figure-3).

Cells of the adrenal gland cortex, zona glomerulosa, of cows with corpora lutea are round and medium-sized. Nuclei are large and chromatin has a net structure. An optimal amount of phospholipids are present, whereas neutral fats are nearly absent. Zona fasciculata is well defined. Phospholipids are evenly distributed in the form of small drops. Angular shaped small cells with dark nuclei predominate in the reticular zone. A-cells, surrounding the wide venous sinuses, occupy the largest part of the brain matter. Glial cell clusters are separated by a connective tissue septum.

**Figure-4:**
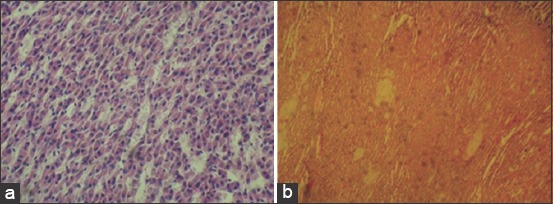
Adrenal gland of an infertile cow with hypofunction of the ovaries. Staining hematoxylin and eosin. (a) Atrophy of endocrinocytes 100× (b) deposition of amyloid in the cortical substance 40×.

The adrenal capsule of cows with hypofunction of the ovaries is well-defined and has a lamellar structure. Zona glomerulosa is narrow, and many large drops of neutral fats are present in its cells. Zona fasciculata is small, and its border with zona glomerulosa is marked. The cells of the reticular zone are located close to each other and the vessels are anemic. Cytoplasmic cells contain vacuoles and pycnosis of cell-nucleus. Degranulation and destruction of A-and N-cells complexes are defined. Amyloid deposits in the adrenal cortex (Figure-4) accompanied by atrophy of the parenchyma were observed.

Dystrophic, atrophic, and necrotic processes in the cortical and cerebral tissues of the adrenal glands of cows with a persistent yellow body were less expressed; however, venous hyperemia and lymphoid infiltrates were specifically attributed to this case. In addition, a distinctive feature was the presence of a small amount of neutral fats and lipochromes in the cortical cells. In zona glomerulosa of cows with follicular cysts, a moderate amount of phospholipids – steroid hormones precursors – and a small amount of neutral lipids were present. Zona fasciculata was well developed and was 3times wider and 1½ times larger than zona glomerulosa and the reticular zone, respectively. Angular shaped small cells with dark nuclei prevailed in the reticular zone. A-cells with a vesicular, eccentrically arranged nucleus occupied 70% of the brain matter volume. Polygonal glial cells were clustered in the brain matter.

## Discussion

Morphological analysis (i.e.,intra vitam tests – cytological and postmortem studies –– and biopsy) is the most accurate method to diagnose diseases of the reproductive organs. No complex morphological studies devoted to functional and morphological analysis of the reproductive system as a whole, including both the study of reproductive organs and the endocrine system, have been mentioned in the literature. Most authors have studied ovaries, oviducts, and the uterus of slaughtered animals [17-21,23,24,34-36]. Some studies conducted pathohistological examination of ovarian follicles and corpus lutea [25,26,29]. In this regard, we attempted to determine clinical and morphological manifestations of the pathology of reproductive organs, especially endocrine glands associated with hypofunction of the ovaries, persistent yellow body, and follicular cysts of ovarian glands of highly productive imported cows. This approach allows to accurately identify pathological processes leading to the infertility of cows [24,25,34-36].

It was clinically and patho-anatomically found that imported cows more often experience ovarian dysfunction accompanied by the pathology of the uterus and oviduct. Hypofunction of the ovaries is diagnosed most often. Moreover, against the background of pathology of other organs, hypofunction of the ovaries can occur in 73% of cows. Suggestive findings of this pathology are long-term anaphrodisia and irreversible atrophy of the uterus and ovaries. Studies have suggested that hypofunction of the ovaries is the most common gynecological disease of highly productive cows. The main causes of ovarian dysfunction are poor nutrition and unfavorable housing conditions [14].

Morphological parameters of the liver and biochemical changes in the blood of cows are the most accurate criteria for differentiating normal and pathological metabolism [37,38]. We studied the metabolism state of lactating cows. The obtained data proved metabolic disorder. Most animals had hypocalcemia and hypocarotinemia. All animals were diagnosed with acidosis. Autopsy proved that 50% of cows experiencing hypofunction of the ovaries presented signs of hepatosis, macroscopically. The association of liver disease with reproductive organs has been emphasized previously [39,40].

We found that the most pronounced hypofunction of the ovaries sign is the absence of the follicle growth and atresia, as well as sclerotic changes in the connective tissue stroma of the organ and in the vascular network. Studies have described the occurrence of same changes in ovaries during hypofunction [41-43].

The state of the uterus and ovaries is certainly closely related to the ovary function. These organs are known to regulate the function of ovarian glands. This was established through histological and histochemical methods in this study – the uterus and oviducts during hypofunction of the ovaries presented signs of atrophic glands and of weakly expressed proliferative processes in the epithelium. Some animals presented morphological signs of endometrium and oviduct inflammation or their complications. Similar changes in the uterus have been described by several authors [44-46].

In our opinion, a systematic approach should be applied to study the reproductive system as a whole. We found that all cell types of endocrinocytes were active in the distal part of adenohypophysis of cycling animals. If persistent corpora lutea are present in the gland, its relative volume is smaller, and it is filled with basophils and acidophils. Morphological signs of these cells are characterized by low activity. During hypofunction of the ovaries, the cellular composition of the distal part of adenohypophysis changes significantly. We noted a decrease in the volumes occupied by chromophobes and basophils; however, the specific volume of acidophils increased significantly. This can explain the decrease in the gonadotropic, TSH, and ACTH hormones secreted by adenohypophyses, which is confirmed by other authors [47].

Activation of acidophilic cells is especially expressed in the ventral zone of the distal part of adenohypophysis. It has been indicated that acidophils of this zone are represented by lactotropocytes (LTG cells; 60%) [47-49]. The role of prolactin in cows is poorly studied. Unlike rats, mice, and sheep, the luteotrophic activity of prolactin has not been found in cattle and pigs [47,50]. The number of LTG cells in the adenohypophysis of cows is significantly influenced by milieu of steroid hormones [51]. An increase in the blood LTG level of cows with anaphrodisia results in a decrease in the pituitary gonadotropic function. Considering laboratory animals and humans, an association between increased prolactin concentration and endocrine infertility has been noted [52].

When generative and endocrine functions of ovaries decrease in cows, pseudofollicles appear in the central zone of adenohypophysis, which are in the state of cystic degeneration. Another characteristic feature of adenohypophysis during hypofunction of the ovaries is an increase in the relative volume of stromal elements, indicating organ atrophy.

During morphological analysis of thyroid glands of cows with hypofunction of the ovaries, we noted signs inherent in the reduction of its functions. This is probably due to the fact that the Republic of Bashkortostan is an endemic zone with a lack of iodine. Thus, the increase in hypothyroidism manifestations depends on the decrease in the endocrine function. Several studies have reported reduced thyroid function during hypofunction of the ovaries [32,53,54].

Marked dystrophic, atrophic, and necrobiotic processes in the adrenal glands during hypofunction of the ovaries and a decrease in their endocrine activity have been observed. This is manifested by a decrease in the number of spongiocytes in the zona fasciculata and in the nuclear-plasma ratio in them, and degranulation of H-and A-cells of the adrenal medulla. All the aforementioned findings correspond to the observations of other researchers [55,56]. In the thyroid and adrenal glands of some infertile cows amyloid was found. The presence of amyloid is related to the development of autoimmune processes in an animal’s body that occurs during chronic forms of metabolic disorders, chronic infections, and purulent necrotic processes in fingers [57,58], which have been diagnosed quite often by us.

## Conclusion

A total of 34% cows imported to the Republic of Bashkortostan were diagnosed with digestive diseases, metabolic disorders, and gynecological diseases.

Hypofunction of the ovaries of sterile highly productive cows is morphologically characterized by intensive atresia of all types of follicles, fibroplastic processes of connective tissue and vasculature, and atrophy of the interstitial gland.

Persistent corpora lutea are morphologically and functionally different from the corpora lutea of cycling animals. Characteristic features are atrophy and fatty degeneration of leukocytes along with an increase in fibrous changes in the connective tissue and vascular hyalinosis.

No signs of secretory activity of the thecal gland in the wall of follicular cysts were observed. However, violations of organ hemodynamics and fibrous and atrophic changes in organ stroma were observed due to large size of these cysts. An increase in the cyst volume followed by luteinization of thecal cells leads to the accumulation of a significant amount of hormonally active luteal tissue, which must be considered when developing methods of diagnosis and treatment of this pathology.

## Authors’ Contributions

ES carried out the study and wrote the manuscript. RM, SB, GB, and VG participated in the drafting and revision of the manuscript. All authors planned and conducted the study. All authors approved the final manuscript.
